# A genetic variant in the promoter of lncRNA MALAT1 is related to susceptibility of ischemic stroke

**DOI:** 10.1186/s12944-020-01236-4

**Published:** 2020-04-01

**Authors:** Yan Wang, Xi-Xi Gu, Hua-Tuo Huang, Chun-Hong Liu, Ye-Sheng Wei

**Affiliations:** 1 Department of Clinical Laboratory, The Affiliated Hospital of Guilin Medical University, Lequn Road No.15, Guilin, 541001 Guangxi Province China; 2 Department of Clinical Laboratory, The Affiliated Hospital of Youjiang Medical University for Nationalities, Baise, Guangxi China

**Keywords:** Ischemic stroke, Polymorphism, Metastasis associated lung adenocarcinoma transcript 1

## Abstract

**Background:**

Metastasis-associated lung adenocarcinoma transcript-1 (*MALAT1*) was aberrantly expressed in diverse diseases. Particularly in ischemic stroke (IS), the abnormal expression of *MALAT1* played important roles including promotion of angiogenesis, inhibition of apoptosis and inflammation and regulation of autophagy. However, the effects of genetic variation (single nucleotide polymorphisms, SNPs) of *MALAT1* on IS have rarely been explored. This study aimed to investigate whether SNPs in promoter of *MALAT1* were associated with the susceptibility to IS.

**Methods:**

A total of 316 IS patients and 320 age-, gender-, and ethnicity-matched controls were enrolled in this study. Four polymorphisms in the promoter of *MALAT1* (i.e., rs600231, rs1194338, rs4102217, and rs591291) were genotyped by using a custom-by-design 48-Plex SNPscan kit.

**Results:**

The rs1194338 C > A variant in the promoter of *MALAT1* was associated with the risk of IS (AC vs. CC: adjusted OR = 0.623, 95% CI, 0.417–0.932, *P* = 0.021; AA vs. CC: adjusted OR = 0.474, 95% CI, 0.226–0.991, *P* = 0.047; Dominant model: adjusted OR = 0.596, 95% CI, 0.406–0.874, *P* = 0.008; A vs. C adjusted OR = 0.658, 95% CI, 0.487–0.890, *P =* 0.007). The haplotype analysis showed that rs600231-rs1194338-rs4102217-rs591291 (A-C-G-C) had a 1.3-fold increased risk of IS (95% CI, 1.029–1.644, *P =* 0.027). Logistic regression analysis identified some independent impact factors for IS including rs1194338 AC/AA, TC, TG, HDL-C, LDL-C, Apo-A1, Apo-B and NEFA (*P* < 0.05).

**Conclusions:**

These results suggest that the rs1194338 AC/AA genotypes may be a protective factor for IS.

## Background

Stroke is a nervous system disease caused by the blood circulation disorder in the brain. It has a high mortality, disability and recurrence rate worldwide. Stroke has become the major cause of death in China, along with approximately 2.5 million new cases and 1.5 million deaths each year [1, 2]. The ischemic stroke (IS) accounts for about 87% of total cases [3]. Therefore, it is urgently required to explore etiology for meaningful targets. As we all known, the IS was a multifactorial complex disease. Traditional factors such as age, gender, obesity, diabetes, hypertension and smoking can only explain part of the IS risk [4–7]. Twins, familial aggregation and genome-wide association studies revealed that heredity was also a powerful factor in progression of IS [8–12].

Long non-coding RNAs (lncRNAs), with a length of more than 200 nucleotides, are emerging as key regulators of physiological and pathological processes [13]. As a highly conserved lncRNA in mammals, *MALAT1* played an important role in cancer. Ghafouri-Fard S et al. revealed that *MALAT1* altered activity or functions of multiple cancer-related signaling pathways such as EMT and PI3K/AKT, but the role of *MALAT1* in carcinogenesis was indefinite, which might be linked with cancer types and sub-types. Moreover, *MALAT1* SNPs modulated the risk of cancer by possibly affecting function or expression levels [14]. Recently, increasing evidence has emerged to support the role of this lncRNA in IS. Using RNA sequencing technology, a lot of lncRNAs with abnormal expression were detected after 16 h under oxygen-glucose deprivation (OGD) condition [15]. *MALAT1*, one of the most highly up-regulated lncRNAs, was further confirmed to promote angiogenesis and autophagy, and reduce apoptosis and inflammation both in vitro and in vivo [16–18]. For example, *MALAT1* reduced ischemic cerebral damages by regulating 15-LOX1, VEGF and STAT3 related to angiogenesis [16]. Moreover, *MALAT1* also acted as a competing endogenous RNA for miR-26b to directly up-regulate the expression of autophagy factor ULK2 to reduce the injury of brain microvascular endothelial cells [17]. Silencing of MALAT1 obviously increased expression of the proapoptotic and proinflammatory cytokines including Bim, IL-6, MCP-1 and E-selectin [18]. Taken together, *MALAT1* appears important not only in cancer development but also in the progression of IS, but the exact mechanism is still not fully elucidated.

Currently, SNPs of lncRNAs have been verified to be associated with IS susceptibility, such as the rs217727 C > T and rs4929984 C > A in lncRNA *H19* and the rs2240183 in promoter of lncRNA *TUG1* [19–21]. It was reported that genetic variants in the promoter region could affect the expression, subcellular localization and structure stability, ultimately affecting progression of relevant diseases [22]. At the same time, studies also showed that *MALAT1* SNPs affected the susceptibility and progression of diseases including hepatocellular cancer, lung adenocarcinoma and pulmonary arterial hypertension [23–25]. However, the impacts of *MALAT1* SNPs on IS is rarely explored. To our knowledge, no study was conducted for association between the SNPs (rs600231, rs1194338, rs591291 and rs4102217) in promoter of *MALAT1* and IS risk. Given the above, we performed SNPs analysis of *MALAT1* from 636 samples (320 controls and 316 IS patients) in Chinese southwestern population, attempting to identify new candidates for the etiology of IS.

## Methods

### Study population

A total of 316 patients with IS and 320 controls were consecutively recruited from the Affiliated Hospital of Youjiang Medical University for Nationalities, Guangxi, China, between March 2015 and July 2018. All of the subjects were native people living in Guangxi province who were unrelated to Han Chinese. The IS patients were diagnosed based on clinical manifestations, physical examination and cranial computed tomography or magnetic resonance imaging. Exclusion criteria were as follows: hemorrhagic stroke, craniocerebral trauma, cardiogenic thrombosis and tumors. Controls matched the cases at the age, and gender frequency were selected from the hospital’s health care center during the same period. Individuals with autoimmune diseases, liver diseases, genetic diseases, blood disorders and tumors were excluded. The clinical data such as age, gender, hypertension, diabetes, smoking status, total cholesterol (TC), triglyceride (TG), high-density lipoprotein cholesterol (HDL-C), low-density lipoprotein cholesterol (LDL-C), apolipoprotein A1 (Apo-A1), apolipoprotein B (Apo-B) and non-esterified fatty acid (NEFA) were collected from our medical records. The interval time was within 24 h between IS onset and biochemical test.

### SNPs selection

(I) The selection criteria for SNP are as follows: (i) tagSNPs in lncRNA *MALAT1*; (ii) the SNPs in promoter region of *MALAT1*; (iii) in silico analysis predicted potentially functional SNPs in the promoter region of *MALAT1*; (iv) the frequency of minor allele > 5% in Chinese Han population. (v) the functional SNPs have been identified in published literature. Finally, four SNPs of rs600231 A > G, rs1194338 C > A, rs4102217 G > C and rs591291 C > T were selected for further analysis.

### Genotyping

Genomic DNA was extracted from mononuclear cells of peripheral blood by a salting-out method. About 3-5 ml blood samples was taken into ethylene diamine tetraacetic acid tubes from each subject before treatment. Genotyping was performed on an ABI 3500 Genetic Analyzer (Applied Biosystems, CA, USA) using the custom-by-design 48-Plex SNPscan kit (Genesky Biotechnologies Inc., Shanghai, China). Genotypic primers for rs600231, rs1194338, rs4102217and rs591291 were showed in Table 1. Meanwhile, about 10% of all samples were selected at random for Sanger sequencing and reached a 100% consistent rate.

**Table 1:** The primer sequences used for detecting four SNPs of the *MALAT1*

SNPs	allele A1	allele A2	universal primer
rs600231	5′-TGAAACCCAGCAGACAGGACT-3’	5′-TGAAACCCAGCAGACAGGACC-3’	5′-GTCACTTCACAGAGAGCTGAGGGC-3’
rs1194338	5′-GGCTCCAGGGCCGTAGATCAA-3’	5′-GGCTCCAGGGCCGTAGATCAC-3’	5′-GGATCTCTCAGAAGCTTGTCTCTTGA-3’
rs4102217	5′-CCTGCTGCCTCCCTTCCTGTG-3’	5′-CCTGCTGCCTCCCTTCCTGTC-3’	5′-CAGCACTTCTGTCAGTCTCTCCAA-3’
rs591291	5′-CCCTCACCCCCGGGTCTGTG-3’	5′-CCCTCACCCCCGGGTCTGTA-3’	5′-GAACCTGTATCCATGGCTTGTTTTT-3’

*SNPs* Single nucleotide polymorphisms

### Statistical analysis

The Student’s t-test was used to compare continuous data (Mean ± SD) such as clinical data from IS cases and controls. The chi-squared test was chosen to analyze Hardy-Weinberg equilibrium (HWE) and categorical data (proportions) such as sex, hypertension, diabetes mellitus and smoker data. Logistic regression was used to assess the risk of IS by odds ratios (OR), 95% confidence intervals (CIs) and *P* value after adjustment of age, gender, diabetes mellitus, hypertension, smoker, TC, TG, HDL-C, LDL-C, Apo-A1, Apo-B and NEFA. Linkage disequilibrium (LD) and haplotype analysis were carried out by SHEsis software (http://analysis.bio-x.cn/myAnalysis.php). The SPSS statistical software package version 20.0 (SPSS, Chicago, USA) was used for all of the statistical analysis. The *P <* 0.05 was considered statistically significant.

## Results

### Characteristics of the study population

The results are exhibited in Table 2. No significant difference was observed in distributions of age and gender between cases and controls. The frequencies of hypertension, diabetes mellitus and smoker in IS patients were obviously higher than those in controls (*P* < 0.05). In addition, IS patients displayed higher levels of TC, TG, LDL-C and Apo-B, and lower levels of HDL-C, Apo-A1, and NEFA (all *P* < 0.05).

**Table 2:** Clinical characteristics of the study population

Variables	Controls, *n* = 320	IS patients, *n* = 316	*P* value
Age, years (Mean ± SD)	60.72 ± 10.77	62.23 ± 11.36	0.087
Gender (M / F)	204 / 116	216 / 100	0.220
Hypertension (%)	59 (18.4%)	126 (39.9)	**< 0.001**
Diabetes mellitus (%)	30 (9.4%)	50 (15.8%)	**< 0.001**
Smoker (%)	50 (15.6%)	98 (31.0%)	**< 0.001**
TCH (mmol/L)	4.19 ± 0.79	4.64 ± 1.16	**< 0.001**
TG (mmol/L)	1.37 ± 0.99	1.81 ± 1.31	**< 0.001**
HDL-C (mmol/L)	1.50 ± 0.31	1.13 ± 0.32	**< 0.001**
LDL -C (mmol/L)	2.39 ± 0.66	2.92 ± 0.98	**< 0.001**
Apo-A1(g/L)	1.73 ± 1.10	1.23 ± 0.26	**< 0.001**
Apo-B (g/L)	0.77 ± 0.31	1.00 ± 0.31	**< 0.001**
NEFA (mmol/L)	0.71 ± 0.30	0.53 ± 0.28	**< 0.001**

*IS* ischemic stroke, *SD* Standard deviation, *M* Male, *F* Female, *TC* Total cholesterol, *TG* Triglyceride, *HDL-C* High density lipoprotein-cholesterol, *LDL-C* Low density lipoprotein-cholesterol, *Apo-A1* Apolipoprotein A1, *Apo-B* Apolipoprotein B, *NEFA* Non-esterified fatty acid

### Association between *MALAT1* polymorphisms and IS risk

The analysis of *MALAT1* SNPs for IS risk is revealed in Table 3. The genotype distributions in controls conformed to HWE (*P* = 0.780 for rs600231, *P* = 0.858 for rs1194338, *P* = 0.569 for rs4102217, *P* = 0.582 for rs591291). Among these SNPs of *MALAT1*, the rs1194338 AC, AA and AC/AA genotype decreased the risk of IS with adjusted OR of 0.623, 0.474 and 0.596, respectively (AC vs. CC: 95% CI, 0.417–0.932, *P* = 0.021; AA vs. CC: 95% CI, 0.226–0.991, *P* = 0.047; AC/AA vs. CC: 95% CI, 0.406–0.874, *P* = 0.008). Similarly, the risk of IS in carriers with A allele was lower than C allele (AOR = 0.658, 95% CI, 0.487–0.890, *P* = 0.007). No significant association was found between other SNPs (rs600231, rs4102217, rs591291) and IS risk.

**Table 3:** Association between the *MALAT1* polymorphisms and risk of IS

SNPs	Controls *n* = 320 (n%)	IS patient *n* = 316 (n%)	AOR† (95%CI)	*P*† value
rs600231				
AA	117 (36.6)	120 (38.0)	1.000 (ref)	
AG	151 (47.2)	154 (48.7)	0.987 (0.651–1.496)	0.950
GG	52 (16.2)	42 (13.3)	0.778 (0.434–1.395)	0.399
Dominant			0.933 (0.629–1.386)	0.733
Recessive			1.276 (0.750–2.171)	0.369
A	385 (60.2)	394 (62.3)	1.000 (ref)	
G	255 (39.8)	238 (37.7)	0.906 (0.688–1.192)	0.481
rs1194338				
CC	154 (48.1)	188 (59.5)	1.000 (ref)	
AC	135 (42.2)	106 (33.5)	0.623 (0.417–0.932)	**0.021**
AA	31 (9.7)	22 (7.0)	0.474 (0.226–0.991)	**0.047**
Dominant			0.596 (0.406–0.874)	**0.008**
Recessive			1.721 (0.841–3.523)	0.137
C	443 (69.2)	482 (76.3)	1.000 (ref)	
A	197 (30.8)	150 (23.7)	0.658 (0.487–0.890)	**0.007**
rs4102217				
GG	243 (75.9)	237 (75.0)	1.000 (ref)	
CG	73 (22.8)	69 (21.8)	1.186 (0.753–1.868)	0.463
CC	4 (1.3)	10 (3.2)	2.322 (0.605–8.906)	0.219
Dominant			1.254 (0.807–1.947)	0.314
Recessive			0.452 (0.119–1.721)	0.244
G	559 (87.3)	543 (85.9)	1.000 (ref)	
C	81(12.7)	89 (14.1)	1.280 (0.869–1.886)	0.212
rs591291				
CC	123 (38.5)	129 (40.8)	1.000 (ref)	
CT	147 (45.9)	144 (45.6)	0.916 (0.607–1.384)	0.678
TT	50 (15.6)	43 (13.6)	0.752 (0.421–1.343)	0.336
Dominant			0.873 (0.592–1.289)	0.495
Recessive			1.268 (0.743–2.163)	0.384
C	393 (61.4)	402 (63.6)	1.000 (ref)	
T	247 (38.6)	230 (36.4)	0.877 (0.665–1.155)	0.350

*IS* Ischemic stroke, *OR* Odds ratio, *95% CI*, 95% confidence interval, *†* Adjusted by age, gender, hypertension, diabetes mellitus, smoker, TCH, TG, HDL-C, LDL-C, Apo-A1, Apo-B, NEFA

### Haplotype analysis of *MALAT1* polymorphisms with IS risk

To further estimate the association between *MALAT1* polymorphism and risk of IS, we performed haplotype analysis. The analysis showed that there was linkage disequilibrium among the four loci, among which the rs600231 and rs591291 showed a strong linkage disequilibrium (D’ = 0.94, r^2^ = 0.83). As summarized in Table 4, possible four haplotypes were listed, and the rs600231-rs1194338-rs4102217-rs591291 (A-C-G-C) haplotype had a 1.3-fold increased risk of IS (95% CI, 1.029–1.644, *P* = 0.027).

**Table 4:** Haplotype analysis of the *MALAT1* polymorphisms with risk of IS

Haplotype	Controls (n %)	IS (n %)	OR (95%CI)	*P* value
ACGC	351 (54.9)	373 (59.1)	1.301 (1.029–1.644)	**0.027**
GAGT	91 (15.3)	86 (13.6)	0.903 (0.659–1.238)	0.527
GACT	71(11.1)	57 (9.1)	0.822 (0.569–1.187)	0.296
GCGT	61(9.6)	62 (9.9)	1.076 (0.742–1.561)	0.699

*IS* Ischemic stroke, *OR* Odds ratio, *95% CI* 95% confidence interval. Only frequency greater than 1% is listed

### Multiple logistic regression analysis

As shown in Table 5, the rs1194338 AC/AA affected the IS risk together with blood lipid index. The specific data were as follows: TC (OR = 1.607; 95%CI, 1.356–1.903), TG (OR = 1.482; 95%CI, 1.242–1.770), HDL-C (OR = 0.020; 95%CI, 0.010–0.040), LDL-C (OR = 2.181; 95%CI, 1.764–2.697), Apo-A1 (OR = 0.006; 95%CI, 0.002–0.013), Apo-B (OR = 23.315; 95%CI, 11.576–46.959), NEFA (OR = 0.092; 95%CI, 0.048–0.177) and rs1194338AC/AA (OR = 0.632; 95%CI, 0.461–0.865) (all *p* < 0.05).

**Table 5:** Logistic regression analysis for independent factors of IS susceptibility

Variables	B	S.E	*P* value	OR (95%CI)
TC	0.47	0.086	**< 0.001**	1.607 (1.356–1.903)
TG	0.39	0.090	**< 0.001**	1.482 (1.242–1.770)
HDL-C	−3.89	0.345	**< 0.001**	0.020 (0.010–0.040)
LDL-C	0.78	0.108	**< 0.001**	2.181 (1.764–2.697)
Apo-A1	−5.193	0.429	**< 0.001**	0.006 (0.002–0.013)
Apo-B	3.149	0.357	**< 0.001**	23.315 (11.576–46.959)
NEFA	−2.389	0.334	**< 0.001**	0.092 (0.048–0.177)
rs1194338AC/AA	−0.459	0.160	**0.004**	0.632 (0.461–0.865)

*TC* Total cholesterol, *TG* Triglyceride, *HDL-C* High density lipoprotein-cholesterol, *LDL-C* Low density lipoprotein-cholesterol, *Apo-A1* Apolipoprotein A1, *Apo-B* Apolipoprotein B, *NEFA* Non-esterified fatty acid

### The analysis of *MALAT1* SNPs and blood lipid levels

The association between *MALAT1* SNPs and lipid levels is showed in Table 6. Unfortunately, no evidence of association was observed between SNPs of *MALAT1* and clinical blood lipid levels of IS patients (*P* > 0.05).

**Table 6:** Association between the *MALAT1* SNPs and blood lipid levels in IS

SNPs	TC, mmol/L	TG, mmol/L	HDL-C, mmol/L	LDL-C, mmol/L	Apo-A1, g/L	Apo-B, g/L	NEFA, mmol/L
rs600231							
AA	4.65 ± 1.20	1.79 ± 1.34	1.13 ± .33	2.94 ± 1.03	1.23 ± 0.26	1.00 ± 0.32	0.53 ± 0.28
AG/GG	4.64 ± 1.08	1.83 ± 1.27	1.12 ± .31	2.88 ± 0.90	1.23 ± 0.25	1.00 ± 0.30	0.55 ± 0.28
t	0.090	−0.245	0.384	0.462	−0.138	0.007	−0.620
p	0.928	0.806	0.701	0.645	0.890	0.995	0.535
rs1194338							
CC	4.68 ± 1.11	1.79 ± 1.38	1.14 ± 0.32	2.94 ± 0.97	1.23 ± 0.23	0.99 ± 0.30	0.52 ± 0.27
AC/AA	4.62 ± 1.19	1.82 ± 1.27	1.12 ± 0.32	2.90 ± 0.99	1.23 ± 0.28	1.01 ± 0.31	0.54 ± 0.29
t	0.478	−0.170	0.519	0.434	−0.032	−0.382	0.498
p	0.633	0.865	0.604	0.664	0.974	0.703	0.619
rs4102217							
GG	4.52 ± 1.21	1.72 ± 1.20	1.12 ± 0.30	2.80 ± 0.99	1.21 ± 0.26	0.98 ± 0.30	0.55 ± 0.29
CG/CC	4.68 ± 1.14	1.83 ± 1.35	1.13 ± 0.33	2.96 ± .98	1.24 ± 0.26	1.01 ± 0.31	0.53 ± 0.28
t	−1.079	−0.677	−0.374	−1.274	−0.642	− 0.861	0.562
p	0.281	0.499	0.708	0.204	0.522	0.390	0.574
rs591291							
CC	4.64 ± 1.22	1.82 ± 1.40	1.13 ± 0.33	2.93 ± 1.05	1.23 ± 0.27	1.00 ± 0.32	0.53 ± 0.28
CT/TT	4.63 ± 1.07	1.78 ± 1.19	1.12 ± 0.31	2.89 ± 0.88	1.24 ± 0.25	1.00 ± 0.29	0.54 ± 0.28
t	0.072	0.262	0.124	0.460	−0.447	0.083	− 0.550
p	0.943	0.794	0.902	0.646	0.655	0.934	0.583

*TC* Total cholesterol, *TG* Triglyceride, *HDL-C* High density lipoprotein-cholesterol, *LDL-C* Low density lipoprotein-cholesterol, *Apo-A1* Apolipoprotein A1, *Apo-B*, Apolipoprotein B, *NEFA* Non-esterified fatty acid

### Bioinformatics analysis

**Fig. 1:**
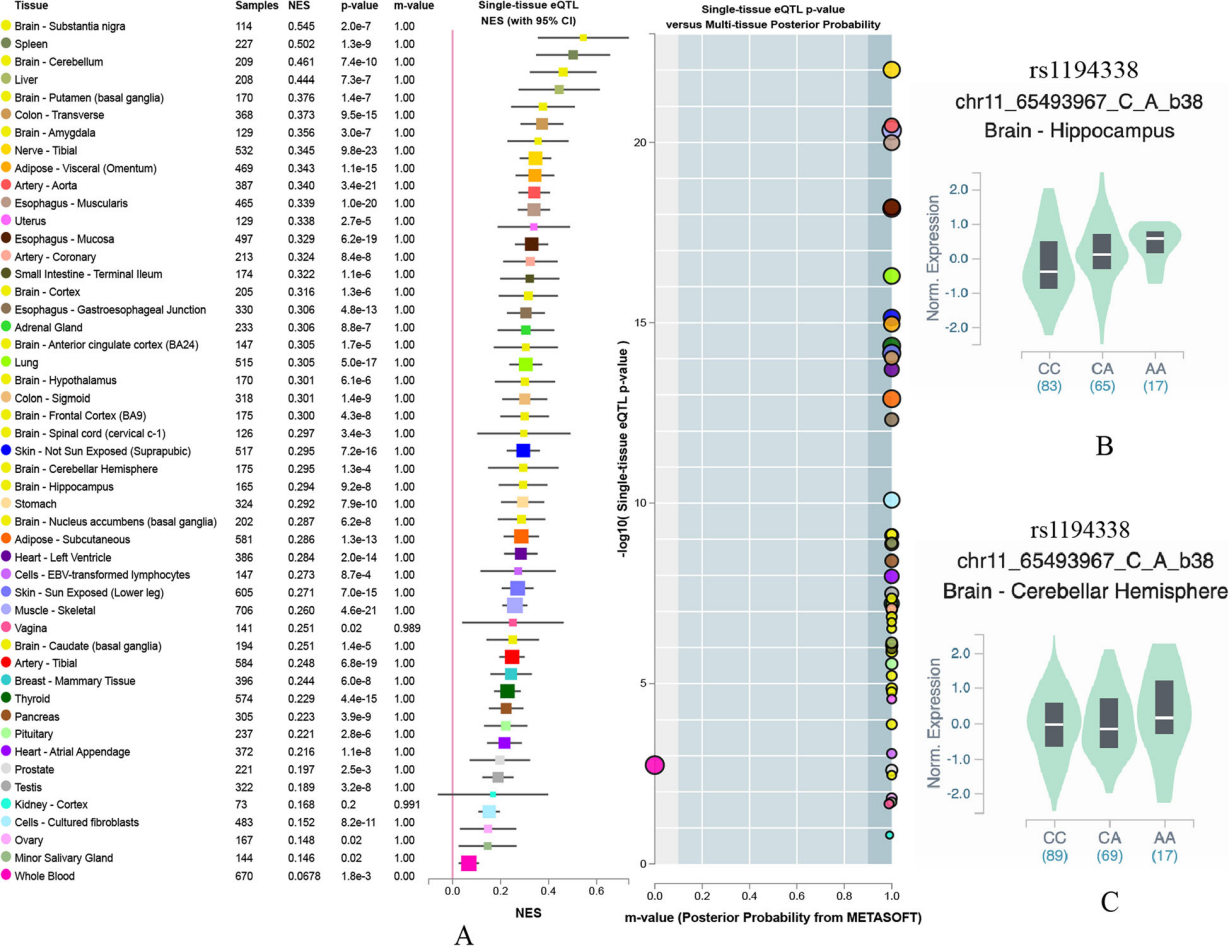
Expression quantitative trait loci (eQTL) anlysis of rs1194338 Expression Quantitative Trait Loci analysis of rs1194338 with gene expression in single tissue (**a**), brain-hippocampus (**b**), and brain-cerebellar hemisphere (**c**)

GTEx data (https://www.gtexportal.org/home/) was used to identify correlations between SNPs and tissue-specific gene expression levels. The analysis of expression quantitative trait loci (eQTL) showed the rs1194338 SNPs were associated with expression of *MALAT1* in single tissue (Fig. 1a), and the carriers with rs1194338 AA increased expression of *MALAT1* in single brain tissue such as brain-hippocampus, brain-cerebellar hemisphere (Fig. 1b-c) (*P* < 0.001).

## Discussion

In the current study, we explored the association between SNPs in the promoter of *MALAT1* and risk of IS. Significant differences were observed in the distribution of the rs1194338 AC/AA genotype and A allele between controls and cases. Further analysis showed that *MALAT1* rs1194338 A allele, AA, AC genotype and the dominant model were associated with decreased risk of IS. Regression analysis revealed that rs1194338AC/AA was one of impact factors of IS together with the lipid index such as TC, TG, HDL-C. In addition, haplotype analysis showed that the ACGC haplotype increased 1.3-fold risk of IS. These findings implicate that *MALAT1* rs1194338 may played the role in the etiology of IS.

*MALAT1*, an 8.1 kb lncRNA, located on human chromosome 11q13. In 2003, Ji et al. first discovered and named *MALAT1* from lung cancer cells [26]. Subsequently, *MALAT1* was confirmed to be associated with tumors cell proliferation, metastasis, survival, and recurrence [27]. New evidence showed that *MALAT1* was abundantly expressed in vascular endothelial cells and participated in processes of neuroprotection of IS by improvement of cognitive function, promotion of angiogenesis, inhibition of apoptosis and inflammation, regulation of autophagy, and protection of blood-brain barrier function [16–18, 28, 29]. The PI3K/AKT pathway was implicated in cell proliferation, apoptosis, and survival under physiological and pathological conditions [30]. The previous study showed a neuroprotective role of early activation of PI3K in ischemic stroke [31]. The result from Yuan et al. demonstrated that overexpression of *MALAT1* decreased cell apoptosis by activating of PI3K/AKT pathway, eventually protect human cerebrovascular endothelial cells in OGD and reoxygenation condition [32]. The above indicate that the *MALAT1* plays a critical role in ischemic stroke, and its high expression may contribute to the protection against brain injury.

In recent years, the association between lncRNA related polymorphisms and risk of diseases has become hotpot of research. As a lncRNA with a wide range of functions, *MALAT1* related SNPs attracted the attention of researchers. For instance, Eftekharian et al. explored the relationship between two *MALAT1* SNPs (rs619586 and rs3200401) and multiple sclerosis (MS) in an Iranian population, and confirmed the G allele of rs619586 significantly reduced the risk of MS with OR of 0.65 [33]. This means that functional SNPs of *MALAT1* may serve as a potential indicator for relevant diseases. The ischemic stroke is one of the diseases threatening human health, and the pathogenesis of IS remain to be fully understood. Actually, increasing studies focused on SNPs of lncRNA involved in process of IS. For example, the rs2240183 C allele of lncRNA *TUG1* was associated with a higher risk of IS by possibly binding to GATA-1 and elevating *TUG1* levels [19]. The lncRNA *ANRIL* rs2383207 increased the risk of IS by 1.52-fold under the recessive model [20]. Furthermore, the rs217727 TT and rs4929984 AA in the lncRNA *H19* increased the risk of IS, with adjusted OR of 4.288, 3.020, respectively [21]. Those studies provided a new perspective on the genetic mechanism of IS. Given above, we hypothesized that the *MALAT1* SNPs were associated with IS risk. Our results supported the above assumption. As shown in Table 3, case-control studies indicated the rs1194338 A allele, AC and AA genotype of *MALAT1* contributed to decreasing risk of IS. Additionally, the ACAG haplotype increased risk of IS (Table 4). Logistic regression also manifested the effect of the rs1194338 AC/AA on risk of IS (Table 5).

The rs1194338, a functional site, located in the promoter region of the *MALAT1*. Recently, several studies indicated the relationship between rs1194338 variant and human diseases. In hepatocellular carcinoma (HCC), female patients and patients with a smoking habit who carried the CA + AA genotype of rs1194338 had a lower risk of developing vascular invasion and a higher Child-Pugh grade, respectively [34]. This suggested there was an interactive function between rs1194338 and the environment. Whether the rs1194338 interacts with the environment in IS remains to be further explored. In colorectal cancer, previous studies found carriers with AA and AC genotype of the rs1194338 were lower risk than CC genotype, and the conclusion from Li’s study showed no statistically significant difference in expression of *MALAT1* between CC and AA genotype at rs1194338 [35, 36]. However, the GTEx database showed rs1194338 SNPs had differences in expression of *MALAT1*. Particularly, the AA genotype of rs1194338 significantly increased expression of *MALAT1* compared to the CC genotype in brain-hippocampus and cerebellar hemisphere tissues (*P < 0.001*) (Fig. 1). Based on the above background, we speculated that the rs1194338 AC/AA genotype might increase the expression of *MALAT1*, which activated related pathways such as PI3K/AKT, thereby decreasing the risk of IS. Further studies are needed to investigate the correlation between the rs1194338 SNPs and expression of *MALAT1* and the precise mechanism of rs1194338 SNPs in IS.

To our knowledge, the study of rs600231 A > G variant with risk of diseases have been rarely reported, but rs4102217 and rs591291 SNPs were evaluated in diseases. Zhang et al. indicated rs4102217 and rs591291 SNPs were not associated with susceptibility of rheumatoid arthritis [37]. The Study of association with HCC have shown rs4102217 had a 1.32-fold risk in the dominant model, and rs591291 highlighted better prognosis in female and HBV negative subgroups, but association between *MALAT1* haplotype (rs4102217-rs591291-rs11227209- rs619586) and HCC risk were not observed [23]. In our study, we found that the ACAG haplotype had a 1.3-fold risk of IS although SNPs (rs600231, rs4102217, rs591291) did not correlate with the susceptibility of IS. It is well known that alteration in blood lipid levels is one of the risk factors in atherosclerotic plaques formation. Atherosclerotic plaques caused easily hypoxia, and possibly resulted in severe diseases such as ischemic stroke [38]. According to the report, *MALAT1* was involved in lipid metabolism [39]. Thus, we further analyzed the relationship between the SNPs of *MALAT1* and blood lipid levels in IS patients. Unfortunately, we failed to observe the significant association. These findings will help improve our understanding of the role of *MALAT1* genetic variants in the pathogenesis of IS.

Although the results we got were promising, limitations still remained. Firstly, a relatively small sample may limit the authenticity of the statistical analysis. Secondly, our study is hospital-based case-control study, and potential selection bias may exist. In addition, the population we studied came from the southwest of China. There are distribution differences in polymorphisms of the same locus among different races according to the 1000 Genomes Project Data. Therefore, larger sample sizes from other medical centers of different races and ethnicities are needed to further confirm the role of *MALAT1* SNPs in IS susceptibility. Finally, the effects of *MALAT1* SNPs on IS are very interesting, but its mechanism is unclear. Both RNA and DNA should be collected simultaneously from the same samples to further verify the effects of SNPs on expression of *MALAT1*.

## Conclusions

In conclusion, our study provides a link between rs1194338 SNPs in promoter of *MALAT1* and the risk of IS, helping to explore the potential molecular mechanisms of IS. In the future, large-scale samples study can be performed among different populations.

## Data Availability

The datasets supporting the conclusions of this article are included within the article.

## Abbreviations

Apo-A1: Apolipoprotein A1
ApoB: Apolipoprotein B
CIs: Confidence intervals
eQTL: Expression Quantitative Trait Loci
F: Female
HCC: Hepatocellular carcinoma
HDL-C: High-density lipoprotein cholesterol
HWE: Hardy-Weinberg equilibrium
IS: Ischemic stroke
LD: Linkage disequilibrium
LDL-C: Low-density lipoprotein cholesterol
lncRNAs: long non-coding RNAs
M: Male
MALAT1: Metastasis-associated lung adenocarcinoma transcript-1
MS: Multiple sclerosis
NEFA: Non-esterified fatty acid
OGD: Oxygen-glucose deprivation
OR: Odds ratio
SD: Standard deviation
SNP: Single nucleotide polymorphism
TC: Total cholesterol
TG: Triglyceride
